# Boundedness of Marcinkiewicz integrals with rough kernels on Musielak-Orlicz Hardy spaces

**DOI:** 10.1186/s13660-017-1501-1

**Published:** 2017-09-19

**Authors:** Bo Li, Minfeng Liao, Baode Li

**Affiliations:** College of Mathematics and System Sciences, Xinjiang University, Urumqi, 830046 P.R. China

**Keywords:** 42B20, 42B30, 46E30, Marcinkiewicz integral, Muckenhoupt weight, Musielak-Orlicz function, Hardy space

## Abstract

Let $\varphi:\mathbb{R}^{n}\times[0, \infty) \to[0, \infty)$ satisfy that $\varphi(x, \cdot)$, for any given $x\in\mathbb{R}^{n}$, is an Orlicz function and $\varphi(\cdot, t)$ is a Muckenhoupt $A_{\infty}$ weight uniformly in $t\in(0, \infty)$. The Musielak-Orlicz Hardy space $H^{\varphi}(\mathbb{R}^{n})$ is defined to be the set of all tempered distributions such that their grand maximal functions belong to the Musielak-Orlicz space $L^{\varphi}(\mathbb{R}^{n})$. In this paper, the authors establish the boundedness of Marcinkiewicz integral $\mu _{\Omega}$ from $H^{\varphi}(\mathbb{R}^{n})$ to $L^{\varphi}(\mathbb{R}^{n})$ under weaker smoothness conditions assumed on Ω. This result is also new even when $\varphi(x, t):=\phi(t)$ for all $(x, t)\in\mathbb{R}^{n}\times[0, \infty)$, where *ϕ* is an Orlicz function.

## Introduction

Suppose that $S^{n-1}$ is the unit sphere in the *n*-dimensional Euclidean space ${{\mathbb{R}}^{n}}$ ($n\ge2$). Let Ω be a homogeneous function of degree zero on ${{\mathbb{R}}^{n}}$ which is locally integrable and satisfies the cancelation condition

1.1 $$\begin{aligned} \int_{S^{n-1}}\Omega\bigl(x'\bigr) \,d\sigma \bigl(x'\bigr)=0, \end{aligned}$$

where *dσ* is the Lebesgue measure and $x':=x/{ \vert x \vert }$ for any $x\neq{\mathbf{0}}$. For a function *f* on ${{\mathbb{R}}^{n}}$, the Marcinkiewicz integral $\mu_{\Omega}$ is defined by setting, for any $x\in{{\mathbb{R}}^{n}}$,

$$\mu_{\Omega}(f) (x):= \biggl( \int_{0}^{\infty} \bigl\vert F_{\Omega, t}(f) (x) \bigr\vert ^{2} \frac{dt}{t^{3}} \biggr)^{\frac{1}{2}}, $$

where

$$F_{\Omega, t}(f) (x):= \int_{ \vert x-y \vert \leq t}\frac {\Omega(x-y)}{ \vert x-y \vert ^{n-1}}f(y) \,dy. $$

In 1938, Marcinkiewicz [1] first defined the operator $\mu _{\Omega}$ for $n=1$ and $\Omega(t):=\operatorname{sign}t$. The Marcinkiewicz integral of higher dimensions was studied by Stein [2] in 1958. He showed that if $\Omega\in\operatorname{Lip}_{\alpha}(S^{n-1})$ with $\alpha\in(0, 1]$, then $\mu_{\Omega}$ is bounded on $L^{p}({{\mathbb{R}}^{n}})$ with $p\in(1, 2]$ and bounded from $L^{1}({{\mathbb{R}}^{n}})$ to $WL^{1}({{\mathbb{R}}^{n}})$. In 1962, Benedek et al. [3] proved that if $\Omega\in C^{1}(S^{n-1})$, then $\mu_{\Omega}$ is bounded on $L^{p}({{\mathbb{R}}^{n}})$ with $p\in (1, \infty)$. In 1990, Torchinsky and Wang [4] proved that, if $\Omega\in\operatorname{Lip}_{\alpha}(S^{n-1})$ with $\alpha\in(0, 1]$, then $\mu_{\Omega}$ is bounded on $L^{p}_{\omega}({{\mathbb{R}}^{n}})$ provided that $p\in (1, \infty)$ and $\omega\in A_{p}$, where $A_{p}$ denotes the Muckenhoupt weight class. Notice that all the results mentioned above hold true depending on some smoothness conditions of Ω. However, in 1999, Ding et al. [5] obtained a celebrated result that $\mu_{\Omega}$ is bounded on $L^{p}_{\omega}({{\mathbb{R}}^{n}})$ without any smoothness conditions of Ω, which is presented as follows.

### Theorem A

*Let* $q\in(1, \infty)$, $q':=q/(q-1)$ *and* $\Omega\in L^{q}(S^{n-1})$ *satisfying* (1.1). *If* $\omega^{q'}\in A_{p}$, $p\in(1, \infty)$, *then there exists a positive constant* *C* *independent of* *f* *such that*

$$\bigl\Vert \mu_{\Omega}(f) \bigr\Vert _{L^{p}_{\omega}({{\mathbb {R}}^{n}})}\le C \Vert f \Vert _{L^{p}_{\omega}({{\mathbb{R}}^{n}})}. $$

It is now well known that the Hardy space $H^{p}({{\mathbb{R}}^{n}})$ is a good substitute of the Lebesgue space $L^{p}({{\mathbb{R}}^{n}})$ with $p\in(0, 1]$ in the study for the boundedness of operators and hence, in 2003, Ding et al. [6] discussed the boundedness of $\mu_{\Omega}$ from the weighted Hardy space to the weighted Lebesgue space under $\Omega\in\operatorname{Lip}_{\alpha}(S^{n-1})$. In 2007, Lin et al. [7] proved that $\mu_{\Omega}$ is bounded from the weighted Hardy space to the weighted Lebesgue space under weaker smoothness conditions assumed on Ω, which is called $L^{q}$-Dini type condition of order *α* (see Section 2 for its definition). For more conclusions of $\mu_{\Omega}$, readers are referred to [8–12].

On the other hand, recently, Ky [13] studied a new Hardy space called Musielak-Orlicz Hardy space $H^{\varphi}({{\mathbb{R}}^{n}})$, which generalizes both the weighted Hardy space (see, for example, [14]) and the Orlicz-Hardy space (see, for example, [15, 16]), and hence has a wide generality. For more information on Musielak-Orlicz-type spaces, see [17–24]. We refer the reader to [24] for a complete survey of the real-variable theory of Musielak-Orlicz Hardy spaces. In light of Lin [7] and Ky [13], it is a natural and interesting problem to ask whether $\mu_{\Omega}$ is bounded from $H^{\varphi}({{\mathbb{R}}^{n}})$ to $L^{\varphi}({{\mathbb{R}}^{n}})$ under weaker smoothness conditions assumed on Ω. In this paper we shall answer this problem affirmatively.

Precisely, this paper is organized as follows.

In Section 2, we recall some notions concerning Muckenhoupt weights, growth functions, $L^{q}$-Dini type condition of order *α* and the Musielak-Orlicz Hardy space $H^{\varphi}({{\mathbb{R}}^{n}})$. Then we present the boundedness of Marcinkiewicz integral $\mu_{\Omega}$ from $H^{\varphi}({{\mathbb{R}}^{n}})$ to $L^{\varphi}({{\mathbb{R}}^{n}})$ under weaker smoothness conditions assumed on Ω (see Theorem 2.4, Theorem 2.5 and Corollary 2.6), the proofs of which are given in Section 3.

In the process of the proof of Theorem 2.4, it is worth pointing out that, since the space variant *x* and the time variant *t* appearing in $\varphi(x, t)$ are inseparable, we cannot directly use the method of Lin [7]. We overcome this difficulty via establishing a more subtle estimate for $\mu_{\Omega}(b)$ on the infinite annuluses away from the support set of *b* (see Lemma 3.4), where *b* is a multiple of a $(\varphi,\infty,s)$-atom. Next, by using the estimate of Lemma 3.4, we find a sequence which is convergent, and fortunately, this sequence converges to a number strictly less than 1, then we can use the uniformly lower type *p* property of *φ*. For more details, we refer the reader to the estimate of ${\mathrm{I}_{2}}$ in the proof of Lemma 3.7. On the other hand, notice that the kernel of $\mu_{\Omega}$ may not belong to Schwartz function space, thus, for a tempered distribution $f\in H^{\varphi}({{\mathbb{R}}^{n}})$, $\mu_{\Omega}(f)$ may be senseless. However, we find that $H^{\varphi}({{\mathbb{R}}^{n}})\cap L^{2}({{\mathbb{R}}^{n}})$ is dense in $H^{\varphi}({{\mathbb{R}}^{n}})$ (see Lemma 3.11). Then, for any $f\in H^{\varphi}({{\mathbb{R}}^{n}})\cap L^{2}({{\mathbb {R}}^{n}})$, now $\mu_{\Omega}(f)$ is well defined and $H^{\varphi}-L^{\varphi}$ boundedness of $\mu_{\Omega}(f)$ for any $f\in H^{\varphi}({{\mathbb{R}}^{n}})\cap L^{2}({{\mathbb{R}}^{n}})$ can be obtained, which can be further uniquely extended from $H^{\varphi}({{\mathbb{R}}^{n}})\cap L^{2}({{\mathbb {R}}^{n}})$ to $H^{\varphi}({{\mathbb{R}}^{n}})$ (see the proof of Lemma 3.12 for more details).

Finally, we make some conventions on notation. Let $\mathbb{Z}_{+}:=\{1, 2, \ldots\}$ and $\mathbb{N}:=\{0\}\cup \mathbb{Z}_{+}$. For any $\beta:=(\beta_{1},\ldots,\beta_{n})\in\mathbb{N}^{n}$, let $\vert \beta \vert :=\beta_{1}+\cdots+\beta_{n}$. Throughout this paper the letter *C* will denote a *positive constant* that may vary from line to line but will remain independent of the main variables. The *symbol* $P\lesssim Q$ stands for the inequality $P\le CQ$. If $P\lesssim Q\lesssim P$, we then write $P\sim Q$. For any sets $E, F \subset{{\mathbb{R}}^{n}}$, we use $E^{\complement}$ to denote the set ${{\mathbb{R}}^{n}}\setminus E$, $\vert E \vert $ its *n-dimensional Lebesgue measure*, $\chi_{E}$ its *characteristic function* and $E+F$ the *algebraic sum* $\{x+y: x\in E, y\in F\}$. For any $s\in\mathbb{R}$, $\lfloor s\rfloor$ denotes the unique integer such that $s-1<\lfloor s\rfloor\le s$. If there are no special instructions, any space $\mathcal{X}({{\mathbb {R}}^{n}})$ is denoted simply by $\mathcal{X}$. For instance, $L^{2}({{\mathbb {R}}^{n}})$ is simply denoted by $L^{2}$. For any index $q\in[1, \infty]$, $q'$ denotes the *conjugate index* of *q*, namely, $1/q+1/{q'}=1$. For any set *E* of ${{\mathbb{R}}^{n}}$, $t\in[0, \infty)$ and measurable function *φ*, let $\varphi(E, t):=\int_{E}\varphi(x, t) \,dx$. As usual we use $B_{r}$ to denote the ball $\{x\in{{\mathbb{R}}^{n}}: \vert x \vert < r\}$ with $r\in(0, \infty)$.

## Notions and main results

In this section, we first recall the notion concerning the Musielak-Orlicz Hardy space $H^{\varphi}$ via the grand maximal function, and then present the boundedness of Marcinkiewicz integral from $H^{\varphi}$ to $L^{\varphi}$.

Recall that a nonnegative function *φ* on ${{\mathbb {R}}^{n}}\times[0, \infty)$ is called *Musielak-Orlicz function* if, for any $x\in{{\mathbb{R}}^{n}}$, $\varphi(x, \cdot)$ is an Orlicz function on $[0, \infty)$ and, for any $t\in[0, \infty)$, $\varphi(\cdot, t)$ is measurable on ${{\mathbb{R}}^{n}}$. Here a function $\phi: [0, \infty) \to[0, \infty)$ is called an *Orlicz function* if it is nondecreasing, $\phi(0) = 0$, $\phi(t) > 0$ for any $t\in(0, \infty)$, and $\lim_{t\to\infty} \phi(t) = \infty$.

For an Orlicz function *ϕ*, the most useful tool to study its growth property may be the upper and the lower types of *ϕ*. More precisely, an Orlicz function *ϕ* is said to be of *lower* (resp. *upper*) *type* *p* with $p \in (-\infty, \infty)$ if there exists a positive constant $C:=C_{\phi}$ such that, for any $t\in[0, \infty)$ and $s \in(0, 1]$ (resp. $s \in[1, \infty)$),

$$\phi(st)\le C s^{p}\phi(t). $$

Given a Musielak-Orlicz function *φ* on ${{\mathbb{R}}^{n}}\times [0, \infty)$, *φ* is said to be of *uniformly lower* (resp. *upper*) *type* *p* with $p\in(-\infty, \infty)$ if there exists a positive constant $C:=C_{\varphi}$ such that, for any $x\in{{\mathbb{R}}^{n}}$, $t\in[0, \infty)$ and $s\in(0, 1]$ (resp. $s\in[1, \infty)$),

$$\begin{aligned} \varphi(x, st)\le C s^{p}\varphi(x, t). \end{aligned}$$

The *critical uniformly lower type index* of *φ* is defined by

2.1 $$\begin{aligned} i(\varphi):=\sup\bigl\{ p\in(-\infty, \infty): \varphi\text{ is of uniformly lower type } p \bigr\} . \end{aligned}$$

Observe that $i(\varphi)$ may not be attainable, namely, *φ* may not be of uniformly lower type $i(\varphi)$ (see [22], p.415, for more details).

### Definition 2.1

(i) Let $q\in[1, \infty)$. A locally integrable function $\varphi(\cdot, t): {{\mathbb{R}}^{n}}\to[0, \infty)$ is said to satisfy the *uniform Muckenhoupt condition* $\mathbb{A}_{q}$, denoted by $\varphi\in\mathbb{A}_{q}$, if there exists a positive constant *C* such that, for any ball $B\subset{{\mathbb{R}}^{n}}$ and $t\in(0, \infty)$, when $q=1$,
  $$\frac{1}{ \vert B \vert } \int_{B} \varphi(x, t) \,dx \Bigl\{ \mathop{\operatorname{ess \,sup}}_{y\in B} \bigl[\varphi(y, t)\bigr]^{-1} \Bigr\} \le C $$
  and, when $q\in(1,\infty)$,
  $$\frac{1}{ \vert B \vert } \int_{B}\varphi(x, t) \,dx \biggl\{ \frac{1}{ \vert B \vert } \int_{B}\bigl[\varphi(y, t)\bigr]^{-\frac{1}{q-1}} \,dy \biggr\} ^{q-1} \le C. $$
(ii) Let $q\in(1, \infty]$. A locally integrable function $\varphi (\cdot, t): {{\mathbb{R}}^{n}}\to[0, \infty)$ is said to satisfy the *uniformly reverse Hölder condition* $\mathbb{RH}_{q}$, denoted by $\varphi\in\mathbb{RH}_{q}$, if there exists a positive constant *C* such that, for any ball $B\subset{{\mathbb{R}}^{n}}$ and $t\in(0, \infty)$, when $q\in (1,\infty)$,
  $$\biggl\{ \frac{1}{ \vert B \vert } \int_{B}\bigl[\varphi(x, t)\bigr]^{q} \,dx \biggr\} ^{\frac{1}{q}} \biggl\{ \frac{1}{ \vert B \vert } \int_{B}\varphi(y, t) \,dy \biggr\} ^{-1}\leq C $$
  and, when $q=\infty$,
  $$\Bigl\{ \mathop{\operatorname{ess\,sup}}_{x\in B} \varphi(x, t) \Bigr\} \biggl\{ \frac {1}{ \vert B \vert } \int_{B}\varphi(y, t) \,dy \biggr\} ^{-1}\leq C. $$

Define $\mathbb{A}_{\infty}:=\bigcup_{q\in[1, \infty)} \mathbb{A}_{q}$ and, for any $\varphi\in\mathbb{A}_{\infty}$,

2.2 $$\begin{aligned} q(\varphi):=\inf\bigl\{ q\in[1, \infty): \varphi\in \mathbb{A}_{q}\bigr\} . \end{aligned}$$

Observe that, if $q(\varphi)\in(1, \infty)$, then $\varphi \notin\mathbb{A}_{q(\varphi)}$, and there exists $\varphi\notin \mathbb{A}_{1}$ such that $q(\varphi)=1$ (see, for example, [25]).

### Definition 2.2

[13], Definition 2.1

A function $\varphi: {{\mathbb{R}}^{n}}\times[0, \infty) \to[0, \infty)$ is called a *growth function* if the following conditions are satisfied:

(i) *φ* is a Musielak-Orlicz function;
(ii) $\varphi\in\mathbb{A}_{\infty}$;
(iii) *φ* is of uniformly lower type *p* for some $p\in(0, 1]$ and of uniformly upper type 1.

Suppose that *φ* is a Musielak-Orlicz function. Recall that the *Musielak-Orlicz space* $L^{\varphi}$ is defined to be the set of all measurable functions *f* such that, for some $\lambda\in(0, \infty)$,

$$\int_{{\mathbb{R}}^{n}}\varphi \biggl(x, \frac{ \vert f(x) \vert }{\lambda} \biggr) \,dx< \infty $$

equipped with the Luxembourg-Nakano (quasi-)norm

$$\Vert f \Vert _{L^{\varphi}}:=\inf \biggl\{ \lambda\in (0, \infty): \int_{{\mathbb{R}}^{n}}\varphi \biggl(x, \frac{ \vert f(x) \vert }{\lambda} \biggr) \,dx\le1 \biggr\} . $$

In what follows, we denote by $\mathcal{S}$ the *set of all Schwartz functions* and by $\mathcal{S}'$ its *dual space* (namely, the *set of all tempered distributions*). For any $m\in\mathbb{N}$, let $\mathcal{S}_{m}$ be the *set* of all $\psi\in\mathcal{S}$ such that $\Vert \psi \Vert _{\mathcal{S} _{m}}\le1$, where

$$\begin{aligned} \Vert \psi \Vert _{\mathcal{S}_{m}}:=\sup_{\alpha\in \mathbb{N}^{n}, \vert \alpha \vert \le m+1}\sup _{x\in{{\mathbb {R}}^{n}}}\bigl(1+ \vert x \vert \bigr)^{(m+2)(n+1)} \bigl\vert \partial^{\alpha}\psi (x) \bigr\vert . \end{aligned}$$

Then, for any $m\in\mathbb{N}$ and $f\in\mathcal{S}'$, the *non-tangential grand maximal function* $f^{\ast}_{m}$ of *f* is defined by setting, for all $x\in{{\mathbb{R}}^{n}}$,

2.3 $$\begin{aligned} f^{\ast}_{m}(x) := \sup_{\psi\in\mathcal{S}_{m}} \sup_{ \vert y-x \vert < t, t\in(0, \infty)} \bigl\vert f\ast\psi_{t}(y) \bigr\vert , \end{aligned}$$

where, for any $t\in(0, \infty)$, $\psi_{t}(\cdot):= t^{-n}\psi (\frac{\cdot}{t})$. When

2.4 $$\begin{aligned} m=m(\varphi) := \biggl\lfloor n \biggl(\frac{q(\varphi)}{i(\varphi )}-1 \biggr) \biggr\rfloor , \end{aligned}$$

we denote $f^{\ast}_{m}$ simply by $f^{\ast}$, where $q(\varphi)$ and $i(\varphi)$ are as in (2.2) and (2.1), respectively.

### Definition 2.3

[13], Definition 2.2

Let *φ* be a growth function as in Definition 2.2. The *Musielak-Orlicz Hardy space* $H^{\varphi}$ is defined as the set of all $f\in\mathcal{S}'$ such that $f^{\ast}\in L^{\varphi}$ endowed with the (quasi-)norm

$$\Vert f \Vert _{H^{\varphi}}:= \bigl\Vert f^{\ast}\bigr\Vert _{L^{\varphi}}. $$

Throughout the paper, we always assume that Ω is homogeneous of degree zero and satisfies (1.1).

Recall that, for any $q\in[1, \infty)$ and $\alpha\in(0, 1]$, a function $\Omega\in L^{q}(S^{n-1})$ is said to satisfy the $L^{q}$ *-Dini type condition of order* *α* (when $\alpha=0$, it is called the $L^{q}$ *-Dini condition*) if

$$\int_{0}^{1}\frac{\omega_{q}(\delta)}{\delta^{1+\alpha}} \,d\delta < \infty, $$

where ${\omega_{q}(\delta)}$ is the integral modulus of continuity of order *q* of Ω defined by setting, for any $\delta\in(0, 1]$,

$${\omega_{q}(\delta)}:=\sup_{ \Vert {\gamma} \Vert < \delta} \biggl( \int_{S^{n-1}} \bigl\vert \Omega\bigl({\gamma}x' \bigr)-\Omega \bigl(x'\bigr) \bigr\vert ^{q} \,d\sigma \bigl(x'\bigr) \biggr)^{\frac{1}{q}} $$

and *γ* denotes a rotation on $S^{n-1}$ with $\Vert {\gamma} \Vert :=\sup_{y'\in S^{n-1}} \vert {\gamma }y'-y' \vert $. For any $\alpha, \beta\in(0, 1]$ with $\beta<\alpha$, it is easy to see that if Ω satisfies the $L^{q}$-Dini type condition of order *α*, then it also satisfies the $L^{q}$-Dini type condition of order *β*. We thus denote by $\operatorname{Din}^{q}_{\alpha}(S^{n-1})$ the class of all functions which satisfy the $L^{q}$-Dini type conditions of all orders $\beta<\alpha$. For any $\alpha\in(0, 1]$, we define

$$\operatorname{Din}^{\infty}_{\alpha}\bigl(S^{n-1}\bigr):= \bigcap_{q\ge1}\operatorname{Din}^{q}_{\alpha}\bigl(S^{n-1}\bigr). $$

See [7], pp.89-90, for more properties of $\operatorname{Din}^{q}_{\alpha}(S^{n-1})$ with $q\in[1, \infty]$ and $\alpha\in(0,1]$.

The main results of this paper are as follows, the proofs of which are given in Section 3.

### Theorem 2.4

*Let* $\alpha\in(0, 1]$, $\beta:=\min\{\alpha, 1/2\}$, $p\in ({n}/{(n+\beta)}, 1)$, $q\in(1, \infty]$, *and let* *φ* *be a growth function as in Definition* 2.2. *Suppose that* $\Omega\in L^{q}(S^{n-1})\cap\operatorname{Din}^{1}_{\alpha }(S^{n-1})$. *If*

(i) $q\in(1, 1/p]$ *and* $\varphi^{q'}\in\mathbb {A}_{\frac{p\beta}{n(1-p)}}$, *or*
(ii) $q\in(1/p, \infty]$ *and* $\varphi ^{{1}/{(1-p)}}\in\mathbb{A}_{\frac{p\beta}{n(1-p)}}$,

*then there exists a positive constant* *C* *independent of* *f* *such that*

$$\bigl\Vert \mu_{\Omega}(f) \bigr\Vert _{L^{\varphi}} \leq C \Vert f \Vert _{H^{\varphi}}. $$

### Theorem 2.5

*Let* $\alpha\in(0, 1]$, $\beta:=\min\{\alpha, 1/2\}$, $p\in ({n}/{(n+\beta)}, 1]$, $q\in(1, \infty)$, *and let* *φ* *be a growth function as in Definition* 2.2. *Suppose that* $\Omega\in\operatorname{Din}^{q}_{\alpha}(S^{n-1})$. *If* $\varphi^{q'}\in\mathbb{A}_{(p+\frac{p\beta}{n}-\frac{1}{q})q'}$, *then there exists a positive constant* *C* *independent of* *f* *such that*

$$\bigl\Vert \mu_{\Omega}(f) \bigr\Vert _{L^{\varphi}} \leq C \Vert f \Vert _{H^{\varphi}}. $$

### Corollary 2.6

*Let* $\alpha\in(0, 1]$, $\beta:=\min\{\alpha, 1/2\}$, $p\in({n}/{(n+\beta)}, 1]$, *and let* *φ* *be a growth function as in Definition* 2.2. *Suppose that* $\Omega\in\operatorname{Din}^{\infty}_{\alpha}(S^{n-1})$. *If* $\varphi\in\mathbb{A}_{p(1+\frac{\beta}{n})}$, *then there exists a positive constant* *C* *independent of* *f* *such that*

$$\bigl\Vert \mu_{\Omega}(f) \bigr\Vert _{L^{\varphi}} \leq C \Vert f \Vert _{H^{\varphi}}. $$

### Remark 2.7

(i) It is worth noting that Corollary 2.6 can be regarded as the limit case of Theorem 2.5 by letting $q\rightarrow\infty$.
(ii) Let *ω* be a classical Muckenhoupt weight and *ϕ* be an Orlicz function.
  - When $\varphi(x, t):=\omega(x)\phi(t)$ for all $(x, t)\in{{\mathbb{R}}^{n}}\times[0, \infty)$, we have $H^{\varphi}\equiv H^{\phi}_{\omega}$. In this case, Theorem 2.4, Theorem 2.5 and Corollary 2.6 hold true for the weighted Orlicz Hardy space. Even when $\omega\equiv1$, the above results are also new.
  - When $\varphi(x, t):=\omega(x) t^{p}$ for all $(x, t)\in {{\mathbb{R}}^{n}}\times[0, \infty)$, namely, $H^{\varphi}\equiv H^{p}_{\omega}$, Theorem 2.4, Theorem 2.5 and Corollary 2.6 are reduced to [7], Theorem 1.4, Theorem 1.5 and Corollary 1.7, respectively.
(iii) Theorem 2.4, Theorem 2.5 and Corollary 2.6 jointly answer the question: when $\Omega\in\operatorname{Din}^{q}_{\alpha}(S^{n-1})$ with $q=1$, $q\in(1, \infty)$ or $q=\infty$, respectively, what kind of additional conditions on growth function *φ* and Ω can deduce the boundedness of $\mu_{\Omega}$ from $H^{\varphi}$ to $L^{\varphi}$?

## Proofs of main results

To show Theorem 2.4, Theorem 2.5 and Corollary 2.6, let us begin with some lemmas. Since *φ* satisfies the uniform Muckenhoupt condition, the proofs of (i), (ii) and (iii) of the following Lemma 3.1 are identical to those of Exercises 9.1.3, Theorem 9.2.5 and Corollary 9.2.6 in [26], respectively, the details being omitted.

### Lemma 3.1

*Let* $q\in[1, \infty]$. *If* $\varphi\in\mathbb{A}_{q}$, *then the following statements hold true*:

(i) $\varphi^{\varepsilon}\in\mathbb{A}_{q}$ *for any* $\varepsilon\in(0, 1]$;
(ii) $\varphi^{\eta}\in\mathbb{A}_{q}$ *for some* $\eta\in (1, \infty)$;
(iii) $\varphi\in\mathbb{A}_{d}$ *for some* $d\in(1, q)$ *with* $q\neq1$.

### Lemma 3.2

[13], Lemma 4.5

*Let* $\varphi\in\mathbb{A}_{q}$ *with* $q\in[1, \infty)$. *Then there exists a positive constant* *C* *such that*, *for any ball* $B\subset{{\mathbb{R}}^{n}}$, $\lambda\in(1, \infty)$ *and* $t\in (0, \infty)$,

$$\varphi(\lambda B, t)\le C{\lambda}^{nq}\varphi(B, t). $$

### Definition 3.3

[13], Definition 2.4

Let *φ* be a growth function as in Definition 2.2.

(i) A triplet $(\varphi, q, s)$ is said to be *admissible* if $q\in(q(\varphi), \infty]$ and $s \in[m(\varphi), \infty )\cap\mathbb{N}$, where $q(\varphi)$ and $m(\varphi)$ are as in (2.2) and (2.4), respectively.
(ii) For an admissible triplet $(\varphi, q, s)$, a measurable function *a* is called a $(\varphi, q, s)$ *-atom* if there exists some ball $B\subset {{\mathbb{R}}^{n}}$ such that the following conditions are satisfied:
  - *a* is supported in *B*;
  - $\Vert a \Vert _{L^{q}_{\varphi}(B)}\leq \Vert \chi_{B} \Vert ^{-1}_{L^{\varphi}}$, where
    $$\begin{aligned} \Vert a \Vert _{L_{\varphi}^{q}(B)}:= \textstyle\begin{cases} \sup_{t\in(0, \infty)} [\frac{1}{\varphi (B, t)}\int_{B} \vert a(x) \vert ^{q} \varphi(x, t) \,dx ]^{1/q},&q\in[1, \infty),\\ \Vert a \Vert _{L^{\infty}(B)},&q=\infty; \end{cases}\displaystyle \end{aligned}$$
  - $\int_{{\mathbb{R}}^{n}}a(x)x^{\alpha}\,dx=0$ for any $\alpha \in\mathbb{N}^{n}$ with $\vert \alpha \vert \leq s$.
(iii) For an admissible triplet $(\varphi, q, s)$, the *Musielak-Orlicz atomic Hardy space* $H^{\varphi, q, s}_{\mathrm{at}}$ is defined as the set of all $f \in\mathcal{S}'$ which can be represented as a linear combination of $(\varphi, q, s)$-atoms, that is, $f =\sum_{j} b_{j}$ in $\mathcal{S}'$, where $b_{j}$ for each *j* is a multiple of some $(\varphi, q, s)$-atom supported in some ball ${x_{j}+B_{r_{j}}}$ with the property
  $$\sum_{j}\varphi \bigl({x_{j}+B_{r_{j}}}, \Vert b_{j} \Vert _{L^{q}_{\varphi}({x_{j}+B_{r_{j}}})} \bigr)< \infty. $$
  For any given sequence of multiples of $(\varphi, q, s)$-atoms, $\{b_{j}\}_{j}$, let
  $$\Lambda_{q}\bigl(\{b_{j}\}_{j}\bigr):=\inf \biggl\{ \lambda\in(0, \infty): \sum_{j} \varphi \biggl({x_{j}+B_{r_{j}}}, \frac{ \Vert b_{j} \Vert _{L^{q}_{\varphi}({x_{j}+B_{r_{j}}})}}{\lambda} \biggr)\le1 \biggr\} $$
  and then the (quasi-)norm of $f\in\mathcal{S}'$ is defined by
  $$\Vert f \Vert _{H^{\varphi, q, s}_{\mathrm{at}}}:=\inf \bigl\{ \Lambda_{q} \bigl( \{b_{j}\}_{j} \bigr) \bigr\} , $$
  where the infimum is taken over all admissible decompositions of *f* as above.

We refer the reader to [13] and [24] for more details on the real-variable theory of Musielak-Orlicz Hardy spaces.

### Lemma 3.4

*Let* *b* *be a multiple of a* $(\varphi, \infty, s)$-*atom associated with some ball* $B_{r}$. *Then there exists a positive constant* $C:=C_{\Omega}$ *independent of* *b* *such that*, *for any* $x\in B_{2R}\setminus B_{R}$ *with* $R\in[2r, \infty)$,

$$\bigl\vert \mu_{\Omega}(b) (x) \bigr\vert \le C \Vert b \Vert _{L^{\infty}} \biggl[\ln{\frac{2R+r}{R-r}}+\frac {(R+2r)^{2}}{2(2R+r)^{2}} \biggr]^{\frac{1}{2}}. $$

### Proof

Observe that, since $\operatorname{supp} b \subset B_{r}$, it follows that, for any $y\in B_{r}$ and $x\in B_{2R}\setminus B_{R}$ with $R\in[2r, \infty)$,

3.1 $$\begin{aligned} R-r< \vert x-y \vert < 2R+r. \end{aligned}$$

On the other hand, for any $x\in B_{2R}\setminus B_{R}$ with $R\in [2r, \infty)$, write

$$\begin{aligned} \bigl\vert \mu_{\Omega}(b) (x) \bigr\vert ^{2} &= \int_{0}^{\infty} \biggl\vert \int_{\vert x-y\vert\leq t} \frac{\Omega(x-y)}{ \vert x-y \vert ^{n-1}}b(y) \,dy \biggr\vert ^{2} \frac{dt}{t^{3}} \\ &= \int_{0}^{R-r} \biggl\vert \int_{\vert x-y\vert\leq t} \frac{\Omega(x-y)}{ \vert x-y \vert ^{n-1}}b(y) \,dy \biggr\vert ^{2} \frac{dt}{t^{3}} + \int_{R-r}^{2R+r}\cdots+ \int_{2R+r}^{\infty}\cdots \\ &=:\mathrm{I}_{1}+\mathrm{I}_{2}+\mathrm{I}_{3}. \end{aligned}$$

For $\mathrm{I}_{1}$, from $t\in(0, R-r]$ and (3.1), it follows that $\{y\in{{\mathbb{R}}^{n}}: \vert x-y \vert \le t\} =\emptyset$ and hence $\mathrm{I}_{1}=0$.

For $\mathrm{I}_{2}$, by a spherical coordinates transform and $\Omega\in L^{1}(S^{n-1})$ (see (1.1)), we obtain

$$\begin{aligned} \mathrm{I}_{2} &\le \Vert b \Vert _{L^{\infty}}^{2} \int_{R-r}^{2R+r} \biggl( \int_{S^{n-1}} \int_{0}^{t} \frac{ \vert \Omega(y') \vert }{\rho^{n-1}}\rho^{n-1} \,d\rho \,d\sigma\bigl(y'\bigr) \biggr)^{2} \frac{dt}{t^{3}} \\ &\sim \Vert b \Vert _{L^{\infty}}^{2} \int_{R-r}^{2R+r}\frac{1}{t} \,dt \sim \Vert b \Vert _{L^{\infty}}^{2} \ln{\frac{2R+r}{R-r}}. \end{aligned}$$

For $\mathrm{I}_{3}$, by (3.1), a spherical coordinates transform and $\Omega\in L^{1}(S^{n-1})$ (see (1.1)), we have

$$\begin{aligned} {\mathrm{I}_{3}} &\leq \Vert b \Vert _{L^{\infty}}^{2} \int_{2R+r}^{\infty}\biggl( \int_{B_{2R+r}\setminus B_{R-r}} \frac{ \vert \Omega(y) \vert }{ \vert y \vert ^{n-1}} \,dy \biggr)^{2} \frac{dt}{t^{3}} \\ &= \Vert b \Vert _{L^{\infty}}^{2} \int_{2R+r}^{\infty}\biggl( \int_{S^{n-1}} \int_{R-r}^{2R+r} \frac{ \vert \Omega(y') \vert }{\rho^{n-1}}\rho^{n-1} \,d\rho \,d\sigma\bigl(y'\bigr) \biggr)^{2} \frac{dt}{t^{3}} \\ &\sim \Vert b \Vert _{L^{\infty}}^{2} (R+2r)^{2} \int _{2R+r}^{\infty}\frac{1}{t^{3}} \,dt\sim \Vert b \Vert _{L^{\infty}}^{2} \frac{(R+2r)^{2}}{2(2R+r)^{2}}. \end{aligned}$$

Combining the estimates of ${\mathrm{I}_{1}}$, ${\mathrm{I}_{2}}$ and ${\mathrm{I}_{3}}$, we obtain the desired inequality. This finishes the proof of Lemma 3.4. □

Since *φ* satisfies the uniform Muckenhoupt condition, the proofs of Lemmas 3.5 and 3.6 are identical to those of Corollary 6.2 in [27] and Lemma 4.4 in [7], respectively, the details being omitted.

### Lemma 3.5

*Let* $d\in(1, \infty)$. *Then* $\varphi^{d}\in\mathbb{A}_{\infty}$ *if and only if* $\varphi\in{\mathbb{RH}_{d}}$.

### Lemma 3.6

*For* $q\in[1, \infty)$ *and* $\alpha\in(0, 1]$, *suppose that* Ω *satisfies the* $L^{q}$-*Dini type condition of order* *α*, *and* $\beta :=\min\{\alpha, 1/2\}$. *Let* $b\in L^{\infty}$ *with* $\operatorname{supp}b\subset B_{r}$ *satisfy*

$$\int_{B_{r}}b(x) \,dx=0. $$

(i) *If* $q=1$, *then there exists a positive constant* *C* *independent of* *b* *such that*, *for any* $R\in[2r, \infty)$,
  $$\int_{B_{2R}\setminus B_{R}} \bigl\vert \mu_{\Omega}(b) (x) \bigr\vert \,dx \leq C \Vert b \Vert _{L^{\infty}}R^{n} \biggl( \frac {r}{R} \biggr)^{n+\beta}. $$
(ii) *If* $q\in(1, \infty)$ *and*, *for any* $(x, t)\in{{\mathbb {R}}^{n}}\times[0, \infty)$, $\varphi(x, t)\ge0$, *then there exists a positive constant* *C* *independent of* *b* *such that*, *for any* $R\in[2r, \infty)$ *and* $t\in[0, \infty)$,
  $$\int_{B_{2R}\setminus B_{R}} \bigl\vert \mu_{\Omega}(b) (x) \bigr\vert \varphi(x, t) \,dx \leq C \Vert b \Vert _{L^{\infty}} \bigl[\varphi ^{q'}(B_{2R}, t) \bigr]^{\frac{1}{q'}}R^{\frac{n}{q}} \biggl(\frac {r}{R} \biggr)^{n+\beta}. $$

### Lemma 3.7

*Let* $\alpha\in(0, 1]$, $\beta:=\min\{\alpha, 1/2\}$, $p\in({n}/{(n+\beta)}, 1)$ *and* $q\in(1, \infty]$. *Suppose that* $\Omega\in L^{q}(S^{n-1})\cap\operatorname{Din}^{1}_{\alpha }(S^{n-1})$. *If*

(i) $q\in(1, 1/p]$ *and* $\varphi^{q'}\in\mathbb {A}_{\frac{p\beta}{n(1-p)}}$, *or*
(ii) $q\in(1/p, \infty]$ *and* $\varphi ^{{1}/{(1-p)}}\in\mathbb{A}_{\frac{p\beta}{n(1-p)}}$,

*then there exists a positive constant* *C* *such that*, *for any* $\lambda\in(0, \infty)$ *and multiple of a* $(\varphi, \infty, s)$-*atom* *b* *associated with some ball* $B\subset{{\mathbb{R}}^{n}}$,

$$\int_{{{\mathbb{R}}^{n}}}\varphi \biggl(x, \frac{ \vert \mu _{\Omega}(b)(x) \vert }{\lambda} \biggr) \,dx \le C\varphi \biggl(B, \frac{ \Vert b \Vert _{L^{\infty}}}{\lambda} \biggr). $$

### Proof

Without loss of generality, we may assume that *b* is a multiple of a $(\varphi, \infty, s)$-atom associated with a ball $B_{r}$ for some $r\in(0, \infty)$. For the general case, we refer the reader to the method of proof in [7], Theorem 1.4. We claim that, in either case (i) or (ii) of Lemma 3.7, there exists some $d\in(1, {p\beta}/{[n(1-p)]})$ such that

3.2 $$\begin{aligned} \varphi^{q'}\in\mathbb{A}_{d} \quad \text{and} \quad \varphi^{1/(1-p)}\in \mathbb{A}_{d}. \end{aligned}$$

We only prove (3.2) under case (ii) since the proof under case (i) is similar. By Lemma 3.1(iii) with $\varphi^{1/{(1-p)}}\in\mathbb {A}_{\frac {p\beta}{n(1-p)}}$, we see that there exists some $d\in(1, {p\beta}/{[n(1-p)]})$ such that $\varphi ^{1/(1-p)}\in\mathbb{A}_{d}$. On the other hand, notice that $q'<1/{(1-p)}$, then, by Lemma 3.1(i), we know $\varphi^{q'}\in\mathbb{A}_{d}$, which is wished.

For any $\lambda\in(0, \infty)$, write

$$\begin{aligned} \int_{{{\mathbb{R}}^{n}}}\varphi \biggl(x, \frac{ \vert \mu _{\Omega}(b)(x) \vert }{\lambda} \biggr) \,dx = \int_{B_{2r}}\varphi \biggl(x, \frac{ \vert \mu_{\Omega}(b)(x) \vert }{\lambda} \biggr) \,dx+ \int_{(B_{2r})^{\complement}}\cdots=:\mathrm{I}_{1}+ \mathrm{I}_{2}. \end{aligned}$$

For $\mathrm{I}_{1}$, by the uniformly upper type 1 property of *φ*, Theorem A with $\Omega\in L^{q}(S^{n-1})$ and $\varphi^{q'}\in\mathbb{A}_{d}$, and Lemma 3.2 with $\varphi\in\mathbb{A}_{d}$ (which is guaranteed by Lemma 3.1(i) with (3.2)), we know that, for any $\lambda\in(0, \infty)$,

$$\begin{aligned} {\mathrm{I}_{1}} &\lesssim \int_{B_{2r}} \biggl(1+\frac{ \vert \mu_{\Omega}(b)(x) \vert }{ \Vert b \Vert _{L^{\infty}}} \biggr)^{d} \varphi \biggl(x, \frac{ \Vert b \Vert _{L^{\infty}}}{\lambda} \biggr) \,dx \\ &\lesssim \int_{B_{2r}} \biggl(1+\frac{ \vert \mu_{\Omega}(b)(x) \vert ^{d}}{ \Vert b \Vert ^{d}_{L^{\infty}}} \biggr)\varphi \biggl(x, \frac{ \Vert b \Vert _{L^{\infty}}}{\lambda} \biggr) \,dx \\ &\lesssim\varphi \biggl(B_{2r}, \frac{ \Vert b \Vert _{L^{\infty}}}{\lambda} \biggr)+ \frac{1}{ \Vert b \Vert ^{d}_{L^{\infty}}} \int_{{\mathbb{R}}^{n}} \bigl\vert \mu_{\Omega}(b) (x) \bigr\vert ^{d} \varphi \biggl(x, \frac{ \Vert b \Vert _{L^{\infty}}}{\lambda} \biggr) \,dx \\ &\lesssim\varphi \biggl(B_{2r}, \frac{ \Vert b \Vert _{L^{\infty}}}{\lambda} \biggr)+ \frac{1}{ \Vert b \Vert ^{d}_{L^{\infty}}} \int_{B_{r}} \bigl\vert b(x) \bigr\vert ^{d} \varphi \biggl(x, \frac{ \Vert b \Vert _{L^{\infty}}}{\lambda } \biggr) \,dx \\ &\lesssim\varphi \biggl(B_{r}, \frac{ \Vert b \Vert _{L^{\infty}}}{\lambda} \biggr), \end{aligned}$$

which is wished.

Now we are interested in ${\mathrm{I}_{2}}$. For any $j\in\mathbb {Z}_{+}$, let $E_{j}:=B_{2^{j+1}r}\setminus B_{2^{j}r}$. By Lemma 3.4, we know that, for any $x\in E_{j}$,

$$\bigl\vert \mu_{\Omega}(b) (x) \bigr\vert \lesssim \Vert b \Vert _{L^{\infty}} \biggl[\ln{\frac{2^{j+1}+1}{2^{j}-1}}+ \frac{(2^{j}+2)^{2}}{2(2^{j+1}+1)^{2}} \biggr]^{\frac{1}{2}}. $$

Notice that

$$\biggl[\ln{\frac{2^{j+1}+1}{2^{j}-1}}+\frac {(2^{j}+2)^{2}}{2(2^{j+1}+1)^{2}} \biggr]^{\frac{1}{2}} \rightarrow \biggl(\ln2+\frac{1}{8} \biggr)^{\frac{1}{2}}< 1 \quad \text{as } j\rightarrow\infty, $$

then there exists some $J\in\mathbb{Z}_{+}$ independent of *b* such that, for any $j\in[J+1, \infty)\cap\mathbb{Z}_{+}$,

$$\biggl[\ln{\frac{2^{j+1}+1}{2^{j}-1}}+\frac {(2^{j}+2)^{2}}{2(2^{j+1}+1)^{2}} \biggr]^{\frac{1}{2}}< 1. $$

From this, the uniformly lower type *p* and the uniformly upper type 1 properties of *φ*, Theorem A with $\Omega\in L^{q}(S^{n-1})$ and $\varphi^{q'}\in\mathbb{A}_{d}$, Lemma 3.2 with $\varphi\in\mathbb{A}_{d}$ (which is guaranteed by Lemma 3.1(i) with (3.2)), and Hölder’s inequality, we deduce that, for any $\lambda\in(0, \infty)$,

$$\begin{aligned} {\mathrm{I}_{2}} &= \sum_{j=1}^{J} \int_{E_{j}}\varphi \biggl(x, \frac{ \vert \mu _{\Omega}(b)(x) \vert }{\lambda} \biggr) \,dx + \sum_{j=J+1}^{\infty} \int_{E_{j}}\varphi \biggl(x, \frac{ \vert \mu_{\Omega}(b)(x) \vert }{\lambda} \biggr) \,dx \\ &\lesssim\sum_{j=1}^{J} \int_{E_{j}} \biggl(1+\frac{ \vert \mu _{\Omega}(b)(x) \vert ^{d}}{ \Vert b \Vert ^{d}_{L^{\infty}}} \biggr)\varphi \biggl(x, \frac{ \Vert b \Vert _{L^{\infty}}}{\lambda} \biggr) \,dx \\ &\quad{} +\sum_{j=J+1}^{\infty} \int_{E_{j}}\frac{ \vert \mu_{\Omega}(b)(x) \vert ^{p}}{ \Vert b \Vert ^{p}_{L^{\infty}}}\varphi \biggl(x, \frac{ \Vert b \Vert _{L^{\infty}}}{\lambda} \biggr) \,dx \\ &\lesssim\sum_{j=1}^{J} \biggl[\varphi \biggl(B_{2^{j+1}r}, \frac { \Vert b \Vert _{L^{\infty}}}{\lambda} \biggr)+\varphi \biggl(B_{r}, \frac{ \Vert b \Vert _{L^{\infty}}}{\lambda } \biggr) \biggr] \\ &\quad{} +\sum_{j=J+1}^{\infty} \int_{E_{j}}\frac{ \vert \mu_{\Omega}(b)(x) \vert ^{p}}{ \Vert b \Vert ^{p}_{L^{\infty}}}\varphi \biggl(x, \frac{ \Vert b \Vert _{L^{\infty}}}{\lambda} \biggr) \,dx \\ &\lesssim\varphi \biggl(B_{r}, \frac{ \Vert b \Vert _{L^{\infty}}}{\lambda} \biggr) + \frac{1}{ \Vert b \Vert ^{p}_{L^{\infty}}}\sum_{j=J+1}^{\infty} \int_{E_{j}} \bigl\vert \mu_{\Omega}(b) (x) \bigr\vert ^{p}\varphi \biggl(x, \frac{ \Vert b \Vert _{L^{\infty}}}{\lambda} \biggr) \,dx \\ &\lesssim\varphi \biggl(B_{r}, \frac{ \Vert b \Vert _{L^{\infty}}}{\lambda} \biggr) \\ &\quad{} +\frac{1}{ \Vert b \Vert ^{p}_{L^{\infty}}}\sum_{j=J+1}^{\infty} \biggl( \int_{E_{j}} \biggl[\varphi \biggl(x, \frac{ \Vert b \Vert _{L^{\infty}}}{\lambda} \biggr) \biggr]^{\frac{1}{1-p}} \,dx \biggr)^{1-p} \biggl( \int_{E_{j}} \bigl\vert \mu_{\Omega}(b) (x) \bigr\vert \,dx \biggr)^{p}. \end{aligned}$$

Notice that $\varphi^{1/{(1-p)}}\in\mathbb{A}_{d}\subset\mathbb {A}_{\infty}$ (see (3.2)). By Lemma 3.5, we have $\varphi\in\mathbb{RH}_{\frac {1}{1-p}}$. Thus, from Lemma 3.2 with $\varphi^{1/{(1-p)}}\in\mathbb{A}_{d}$ and $\varphi\in\mathbb{RH}_{\frac{1}{1-p}}$, we deduce that, for any $\lambda\in(0, \infty)$,

$$\begin{aligned} \biggl( \int_{E_{j}} \biggl[\varphi \biggl(x, \frac{ \Vert b \Vert _{L^{\infty}}}{\lambda} \biggr) \biggr]^{\frac{1}{1-p}} \,dx \biggr)^{1-p} &\leq \biggl[ \varphi^{\frac{1}{1-p}} \biggl(B_{2^{j+1}r}, \frac { \Vert b \Vert _{L^{\infty}}}{\lambda} \biggr) \biggr]^{1-p} \\ &\lesssim2^{jnd(1-p)} \biggl[\varphi^{\frac{1}{1-p}} \biggl(B_{r}, \frac{ \Vert b \Vert _{L^{\infty}}}{\lambda} \biggr) \biggr]^{1-p} \\ &\lesssim2^{jnd(1-p)}r^{-np}\varphi \biggl(B_{r}, \frac{ \Vert b \Vert _{L^{\infty}}}{\lambda} \biggr). \end{aligned}$$

Since $d<{p\beta}/{[n(1-p)]}$, we may choose $\widetilde{\alpha}\in (0, \alpha)$ such that $d<{p\widetilde{\beta}}/{[n(1-p)]}$, where $\widetilde {\beta }:=\min\{\widetilde{\alpha}, 1/2\}$. By the assumption $\Omega\in\operatorname{Din}^{1}_{\alpha}(S^{n-1})$, Ω satisfies the $L^{1}$-Dini type condition of order *α̃*. Applying Lemma 3.6(i), we obtain

$$\int_{E_{j}} \bigl\vert \mu_{\Omega}(b) (x) \bigr\vert \,dx \lesssim \Vert b \Vert _{L^{\infty}} \bigl(2^{j}r \bigr)^{n} \biggl(\frac{r}{2^{j}r} \biggr)^{n+\widetilde{\beta}} \thicksim \Vert b \Vert _{L^{\infty}}r^{n} 2^{-j\widetilde {\beta}}. $$

Substituting the above two inequalities back into ${\mathrm{I}_{2}}$, we know that, for any $\lambda\in(0, \infty)$,

$${\mathrm{I}_{2}} \lesssim\varphi \biggl(B_{r}, \frac{ \Vert b \Vert _{L^{\infty}}}{\lambda} \biggr) \Biggl(1+\sum_{j=J+1}^{\infty }2^{j(nd-ndp-p\widetilde{\beta})} \Biggr) \lesssim\varphi \biggl(B_{r}, \frac{ \Vert b \Vert _{L^{\infty}}}{\lambda} \biggr), $$

where the last inequality is due to $d<{p\widetilde{\beta}}/{[n(1-p)]}$.

Combining the estimates of $\mathrm{I}_{1}$ and $\mathrm{I}_{2}$, we obtain the desired inequality. This finishes the proof of Lemma 3.7. □

The following three lemmas come from [13], Lemma 4.1, Lemma 4.3(i) and Theorem 3.1, respectively, and also can be found in [24].

### Lemma 3.8

*Let* *φ* *be a growth function as in Definition* 2.2. *Then there exists a positive constant* *C* *such that*, *for any* $(x, t_{j})\in{{\mathbb{R}}^{n}}\times[0, \infty)$ *with* $j\in \mathbb{Z}_{+}$,

$$\varphi \Biggl(x, \sum_{j=1}^{\infty}t_{j} \Biggr)\le C\sum_{j=1}^{\infty}\varphi (x, t_{j} ). $$

### Lemma 3.9

*Let* *φ* *be a growth function as in Definition* 2.2. *For a given positive constant* *C̃*, *there exists a positive constant* *C* *such that*, *for any* $\lambda\in (0, \infty)$,

$$\int_{{\mathbb{R}}^{n}}\varphi \biggl(x, \frac{ \vert f(x) \vert }{\lambda} \biggr) \,dx\le \widetilde{C}\quad \textit{implies that}\quad \Vert f \Vert _{L^{\varphi}}\le C \lambda. $$

### Lemma 3.10

*Let* $(\varphi, q, s)$ *be an admissible triplet as in Definition* 3.3. *Then*

$$H^{\varphi}=H^{\varphi, q, s}_{{\mathrm{at}}} $$

*with equivalent* (*quasi*-)*norms*.

### Lemma 3.11

[24], Remark 4.1.4(i)

*Let* *φ* *be a growth function as in Definition* 2.2. *Then* $H^{\varphi}\cap L^{2}$ *is dense in* $H^{\varphi}$.

The following lemma gives a criterion of the boundedness of operators from $H^{\varphi}$ to $L^{\varphi}$.

### Lemma 3.12

*Let* *φ* *be a growth function as in Definition* 2.2. *Suppose that a linear or a positive sublinear operator* *T* *is bounded on* $L^{2}$. *If there exists a positive constant* *C* *such that*, *for any* $\lambda\in(0, \infty)$ *and multiple of a* $(\varphi, q, s)$-*atom* *b* *associated with some ball* $B\subset{{\mathbb{R}}^{n}}$,

3.3 $$\begin{aligned} \int_{{{\mathbb{R}}^{n}}}\varphi \biggl(x, \frac{ \vert T(b)(x) \vert }{\lambda} \biggr) \,dx \le C\varphi \biggl(B, \frac{ \Vert b \Vert _{L^{q}_{\varphi}(B)}}{\lambda} \biggr), \end{aligned}$$

*then* *T* *extends uniquely to a bounded operator from* ${H^{\varphi}}$ *to* $L^{\varphi}$.

### Proof

We first assume that $f\in H^{\varphi}\cap L^{2}$. By the well-known Calderón reproducing formula (see also [28], Theorem 2.14), we know that there exists a sequence of multiples of $(\varphi, q, s)$-atoms $\{b_{j}\}_{j\in\mathbb{Z}_{+}}$ associated with balls $\{x_{j}+B_{r_{j}}\}_{j\in\mathbb{Z}_{+}}$ such that

3.4 $$\begin{aligned} f=\lim_{k\rightarrow\infty}\sum_{j=1}^{k} b_{j} =:\lim_{k\rightarrow\infty}f_{k} \quad \text{in } \mathcal{S}' \text{ and also in } L^{2}. \end{aligned}$$

From the assumption that the linear or positive sublinear operator *T* is bounded on $L^{2}$ and (3.4), it follows that

$$\begin{aligned} \lim_{k\rightarrow\infty} \bigl\Vert T(f)-T (f_{k} ) \bigr\Vert _{L^{2}} \le\lim_{k\rightarrow\infty} \bigl\Vert T (f-f_{k} ) \bigr\Vert _{L^{2}}\lesssim\lim _{k\rightarrow\infty} \Vert f-f_{k} \Vert _{L^{2}}=0, \end{aligned}$$

which implies that

$$T(f)=\lim_{k\rightarrow\infty}T (f_{k} ) \le\lim _{k\rightarrow\infty}\sum_{j=1}^{k}T (b_{j} ) =\sum_{j=1}^{\infty}T (b_{j} ) \quad \text{almost everywhere}. $$

By this, Lemma 3.8 and (3.3), we obtain

$$\begin{aligned} \int_{{{\mathbb{R}}^{n}}}\varphi \biggl(x, \frac{ \vert T(f)(x) \vert }{\Lambda_{q}(\{b_{j}\}_{j})} \biggr) \,dx & \lesssim\sum_{j=1}^{\infty} \int_{{{\mathbb{R}}^{n}}}\varphi \biggl(x, \frac { \vert T(b_{j})(x) \vert }{\Lambda_{q}(\{b_{j}\}_{j})} \biggr) \,dx \\ &\lesssim\sum_{j=1}^{\infty}\varphi \biggl(x_{j}+B_{r_{j}}, \frac{ \Vert b_{j} \Vert _{L^{q}_{\varphi}(x_{j}+B_{r_{j}})}}{\Lambda_{q}(\{b_{j}\}_{j})} \biggr)\lesssim1, \end{aligned}$$

which, together with Lemma 3.9, further implies that

$$\bigl\Vert T(f) \bigr\Vert _{L^{\varphi}}\lesssim\Lambda_{q} \bigl(\{b_{j}\}_{j}\bigr). $$

Taking infimum for all admissible decompositions of *f* as above and using Lemma 3.10, we obtain that, for any $f\in H^{\varphi}\cap L^{2}$,

3.5 $$\begin{aligned} \bigl\Vert T(f) \bigr\Vert _{L^{\varphi}} \lesssim \Vert f \Vert _{H^{\varphi, q, s}_{{\mathrm{at}}}} \lesssim \Vert f \Vert _{H^{\varphi}}. \end{aligned}$$

Next, suppose $f\in H^{\varphi}$. By Lemma 3.11, it follows that $H^{\varphi}\cap L^{2}$ is dense in $H^{\varphi}$. From this, (3.5) and a standard density argument, we deduce that *T* extends uniquely to a bounded operator from $H^{\varphi}$ to $L^{\varphi}$, namely, $\Vert T(f) \Vert _{L^{\varphi}}\lesssim \Vert f \Vert _{H^{\varphi}}$. This finishes the proof of Lemma 3.12. □

### Proof of Theorem 2.4

Obviously, $\mu_{\Omega}$ is a positive sublinear operator. From Theorem A with $\omega\equiv1$ and Lemma 3.7, it follows that $\mu_{\Omega}$ is bounded on $L^{2}$ and, for any $\lambda\in(0, \infty)$ and multiple of a $(\varphi, \infty, s)$-atom *b* associated with some ball $B\subset{{\mathbb{R}}^{n}}$,

$$\int_{{{\mathbb{R}}^{n}}}\varphi \biggl(x, \frac{ \vert \mu _{\Omega}(b)(x) \vert }{\lambda} \biggr) \,dx \lesssim\varphi \biggl(B, \frac{ \Vert b \Vert _{L^{\infty}}}{\lambda} \biggr). $$

Applying Lemma 3.12 with $q=\infty$, we know that $\Vert \mu_{\Omega}(f) \Vert _{L^{\varphi}}\lesssim \Vert f \Vert _{H^{\varphi}}$. This finishes the proof of Theorem 2.4. □

### Proof of Theorem 2.5

By using the same method as in Theorem 2.4 and repeating the estimate of *J* in the proof of [7], Theorem 1.5, with [7], Lemma 4.4(a), replaced by Lemma 3.6(ii), it is quite believable that Theorem 2.5 holds true. We leave the details to the interested reader. □

### Proof of Corollary 2.6

By Lemma 3.1(ii) with $\varphi\in\mathbb{A}_{p(1+\frac {\beta }{n})}$, we see that there exists some $d\in(1, \infty)$ such that $\varphi^{d}\in\mathbb{A}_{p(1+\frac{\beta}{n})}$. For any $q\in(1, \infty)$, by $p>n/{(n+\beta)}$, we have $(p+p\beta/n-1/q)q'>p(1+\beta/n)$ and hence $\varphi^{d}\in\mathbb{A}_{(p+\frac{p\beta}{n}-\frac{1}{q})q'}$. Thus, we may choose $q=d/{(d-1)}$ such that

$$\varphi^{q'}=\varphi^{d}\in\mathbb{A}_{(p+\frac{\beta}{n}-\frac{1}{q})q'} $$

and hence Corollary 2.6 follows from Theorem 2.5. □

## Conclusions

What we have seen from the above is the boundedness of Marcinkiewicz integral $\mu_{\Omega}$ from $H^{\varphi}$ to $L^{\varphi}$ under weaker smoothness conditions assumed on Ω, which generalizes the corresponding results under the setting of both the weighted Hardy space (see, for example, [14]) and the Orlicz-Hardy space (see, for example, [15, 16]), and hence has a wide generality.
